# Maternal Metformin Treatment Reprograms Maternal High-Fat Diet-Induced Hepatic Steatosis in Offspring Associated with Placental Glucose Transporter Modifications

**DOI:** 10.3390/ijms232214239

**Published:** 2022-11-17

**Authors:** Chien-Fu Huang, Mao-Meng Tiao, I-Chun Lin, Li-Tung Huang, Jiunn-Ming Sheen, You-Lin Tain, Chien-Ning Hsu, Ching-Chou Tsai, Yu-Ju Lin, Hong-Ren Yu

**Affiliations:** 1 Department of Pediatrics, Kaohsiung Chang Gung Memorial Hospital, Kaohsiung 833, Taiwan; 2 Graduate Institute of Clinical Medical Science, Chang Gung University College of Medicine, Kaohsiung 833, Taiwan; 3 Department of Pharmacy, Kaohsiung Chang Gung Memorial Hospital, Kaohsiung 833, Taiwan; 4 Department of Obstetrics and Gynecology, Kaohsiung Chang Gung Memorial Hospital, Kaohsiung 833, Taiwan

**Keywords:** maternal high-fat, metformin, placenta, steatosis, DOHaD

## Abstract

Maternal high-fat (HF) diet exposure in utero may affect fetal development and cause metabolic problems throughout life due to lipid dysmetabolism and oxidative damage. Metformin has been suggested as a potential treatment for body weight reduction and nonalcoholic fatty liver disease, but its reprogramming effect on offspring is undetermined. This study assesses the effects of maternal metformin treatment on hepatic steatosis in offspring caused by maternal HF diet. Female rats were fed either a control or an HF diet before conception, with or without metformin treatment during gestation, and placenta and fetal liver tissues were collected. In another experiment, the offspring were fed a control diet until 120 d (adult stage). Metformin treatment during pregnancy ameliorates placental oxidative stress and enhances placental glucose transporter 1 (*GLUT1*), *GLUT3*, and *GLUT4* expression levels through 5’ adenosine monophosphate-activated protein kinase (AMPK) activation. Maternal metformin treatment was shown to reprogram maternal HF diet-induced changes in offspring fatty liver with the effects observed in adulthood as well. Further validation is required to develop maternal metformin therapy for clinical applications.

## 1. Introduction

Obesity is a chronic disease and a risk factor for other comorbidities and its prevalence has nearly tripled worldwide since 1975, with over 1.9 billion adults overweight or obese in 2016 [1]. High-fat (HF) diets are associated with increased body weight and risk of obesity in humans and animal models [2,3]. The prevalence of overweight/obesity in women of childbearing age is also increasing worldwide with over 60% of women in the United States being either overweight or obese at conception [4]. Maternal obesity during pregnancy is associated with fetal macrosomia, neural tube defects, childhood obesity, and metabolic problems such as cardiovascular disease, coronary heart disease, type 2 diabetes, and stroke [5,6].

Metformin is a first-line therapy for the treatment of type 2 diabetes due to its robust glucose-lowering effects, well-established safety profile, and relatively low cost [7]. Glucose levels are maintained by the inhibition of hepatic gluconeogenesis [8]. Glucose uptake in skeletal muscle cells and adipocytes is induced by insulin and involves glucose transporters (GLUT) [9]. The action of metformin is also related to the diminished secretion of lipids from intestinal epithelial cells as well as the increased oxidation of fatty acids in muscles and adipose tissue [10,11].

Metformin has recently been used as an adjunct therapy for the prevention of obesity in pregnant women since it improves the metabolic status during pregnancy [12]. Administration of metformin prenatally has also been shown to be effective in preventing overweight in pregnancy but does not affect the birth weight of the newborn [13,14]. However, the long-term effects of prenatal metformin on offspring are unknown. In a previous study, we showed that maternal HF diet exposure in utero causes steatosis in the offspring throughout life as a consequence of lipid dysmetabolism and oxidative damage to the fetal liver due to a remodeled placenta [15]. The aim of this study was to assess the short- and long-term reprogramming effects of maternal metformin treatment on offspring obesity induced by maternal HF diet. The reprogramming mechanism of maternal metformin treatment was also investigated.

## 2. Results

### 2.1. Metformin Treatment during Pregnancy Ameliorates HF Diet-Induced Obesity and Hepatic Steatosis in Dams

**Figure 1:**
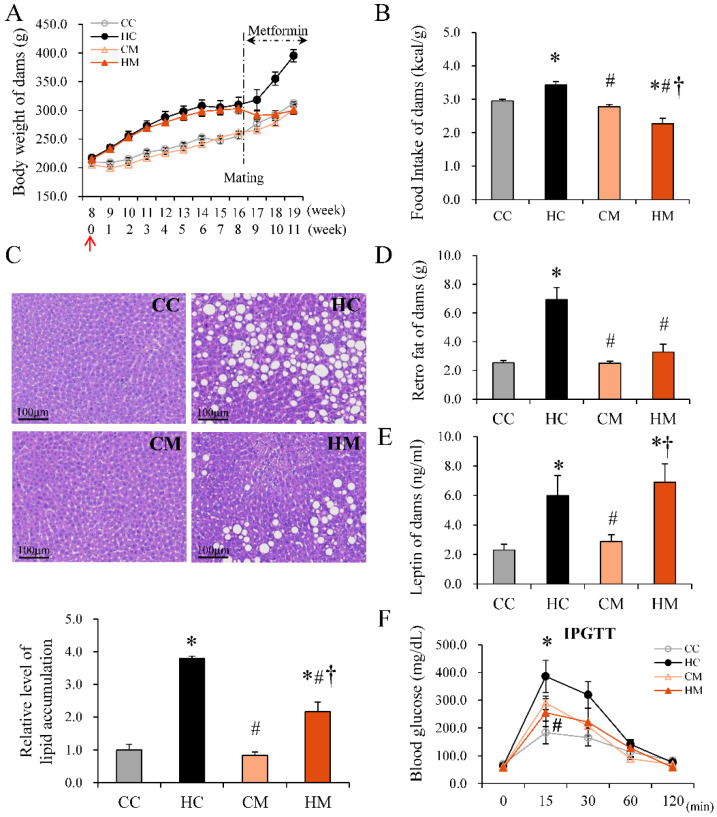
Physiological characteristics of dams fed a control diet (CC), high-fat diet (HC), maternal control diet with maternal metformin treatment after mating (CM), or maternal high-fat diet with maternal metformin treatment after mating (HM). (**A**) Body weight (BW) change. The red arrow indicates the beginning of diet manipulation. The numbers on the upper row represent the age of the dams, and the numbers on the lower row represent the time period of food manipulation. A significant increase in BW was observed after one week of high-fat diet manipulation. Metformin was then administered after mating. A significant decrease in BW was observed after 2 weeks of metformin treatment (#). Detailed BW data are presented in Supplementary Table S2. (**B**) Caloric intake per unit of BW. (**C**) Histological manifestation of liver tissue: The degree of hepatic steatosis is presented as a vacuolation increase via hematoxylin and eosin (H&E) staining. (**D**) Weight of retroperitoneal fat depot. (**E**) Plasma leptin level. (**F**) Data from the intraperitoneal glucose tolerance test (IPGTT). Detailed blood sugar and area under the curve (AUC) data are presented in Supplementary Table S3. The results are presented as the mean ± standard error. * Compared to the CC group, *p* < 0.05; # compared to the HC group, *p* < 0.05; † compared to the CM group, *p* < 0.05.

To investigate the therapeutic effect of maternal metformin, rats were divided into four groups: CC group—dams were fed with a normal chow diet before and during pregnancy. HC group—dams were fed with an HF diet before and during pregnancy. CM group—dams were fed with a normal chow diet before and during pregnancy, and metformin (500 mg/kg/day), dissolved in drinking water, was administered between gestational day (GD) 0 and GD 21. HM group—dams were fed with an HF diet before and during pregnancy, and metformin (500 mg/kg/day), dissolved in drinking water, was administered between gestational day (GD) 0 and GD 21. The body weights (BW) of the dams were determined weekly after the prescribed diet until they were sacrificed (Figure 1A). Similar to our previous study [15], the HF diet significantly increased the BWs of dams compared to the control diet after one week (HC vs. CC; 234.51 ± 5.59 g vs. 208.80 ± 2.68 g; *p* < 0.05), and the BWs continued to increase until the end of the experiment. Metformin was administered from conception. Pregnant dams fed an HF diet after two weeks of metformin treatment demonstrated lower BWs than those without metformin treatment (HM vs. HC; 292.95 ± 6.91 g vs. 355.29 ± 11.52 g; *p* < 0.05). Upon metformin treatment, pregnant dams in the HM group had BWs similar to those of the control diet (CC group). Metformin may have affected the BWs of dams either through decreased intake or metabolic regulation. We found that after 16 weeks of diet manipulation and three weeks of metformin therapy during pregnancy, dams in the HM group showed lower average calorie intake per BW unit than the HC group (HC vs. HM; 3.21 ± 0.52 vs. 2.80 ± 0.52 Kcal/g; *p* < 0.05) (Figure 1B). Thus, metformin seemed to decrease the appetite of pregnant dams on an HF diet. Each dam fed an HF diet showed obvious steatotic changes in the liver, including macrovesicular and microvesiular changes (cytoplasm showed several small vacuoles but the nucleus was not deviated from the center) (Figure 1C). Metformin treatment ameliorated the fatty liver in dams fed an HF diet. It also reduced retroperitoneal fat deposit weight when compared to dams receiving an HF diet without metformin (Figure 1D). Although metformin reduced HF diet-related obesity and retroperitoneal adiposity in dams, it did not affect serum leptin concentration (Figure 1E). An intraperitoneal glucose tolerance test (IPGTT), revealed that dams in the HM group had lower glucose levels at 15 min than the dams in the HC group. The area under the curve (AUC) was marginally lower for dams in the HM group than for those in the HC group, but without statistical significance (Figure 1F).

### 2.2. Metformin Treatment during Pregnancy Decreases Placental Oxidative Stress but Does Not Reverse Placental Remodeling Induced by HF Diet

**Figure 2:**
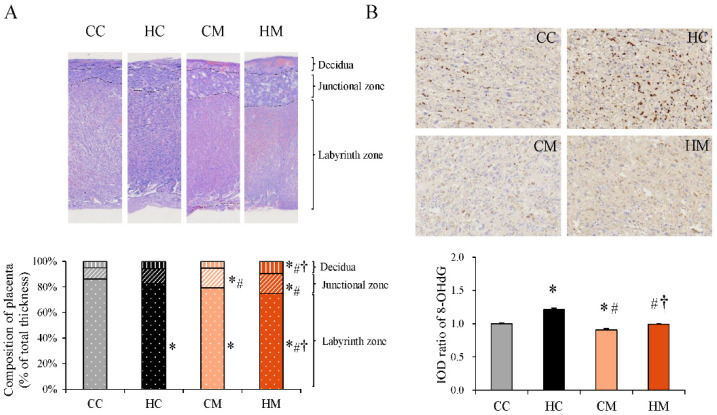
Histological features of placenta with maternal high-fat diet and metformin therapy. (**A**) Histological exhibition of the placenta. The average thicknesses of the basal decidua, junctional zone, and labyrinth zone are illustrated. Magnification at 10× (**B**) Oxidative stress was determined by 8-hydroxy-2-deoxyguanosine (8-OHdG) staining. Magnification at 40× * Compared to the CC group, *p* < 0.05; # compared to the HC group, *p* < 0.05; † compared to the CM group, *p* < 0.05. CC, maternal control diet group; HC, maternal high-fat diet group; CM, maternal control diet with metformin treatment after mating; HM, maternal high-fat diet with metformin treatment after mating; IOD, image optical density.

There were no significant differences in placental weight and litter characteristics among the four groups after sacrifice (Supplementary Figure S1). As the bridge between fetal and maternal circulation, the placenta plays an important role in supplying nutrients to the fetus. The mature placenta consists of three layers of tissue: the outside maternal decidua, the junctional zone, and the inner labyrinth zone. HF diet exposure during pregnancy was found to decrease the thickness of the labyrinth zone [15]. We examined whether metformin treatment during pregnancy can reverse placental remodeling induced by HF diet exposure. We found that metformin treatment during pregnancy did not alter HF diet-induced decreased thickness of the labyrinth zone (Figure 2A). However, the decidua and the junctional zone became thicker after metformin treatment. Maternal metformin treatment alone decreased the placental labyrinth zone as compared with the control group. Oxidative stress, examined by 8-hydroxy-2-deoxyguanosine (8-OHdG) staining, was decreased upon metformin consumption after an HF diet during pregnancy (Figure 2B). Reduced 8-OHdG staining was observed in the maternal metformin-treated group as compared with the staining in the control group.

### 2.3. Maternal HF Diet Intake Decreases the Expression of GLUT4, While Metformin Treatment during Pregnancy Enhances GLUT1, GLUT3, and GLUT4 Expression Levels in the Placenta

**Figure 3:**
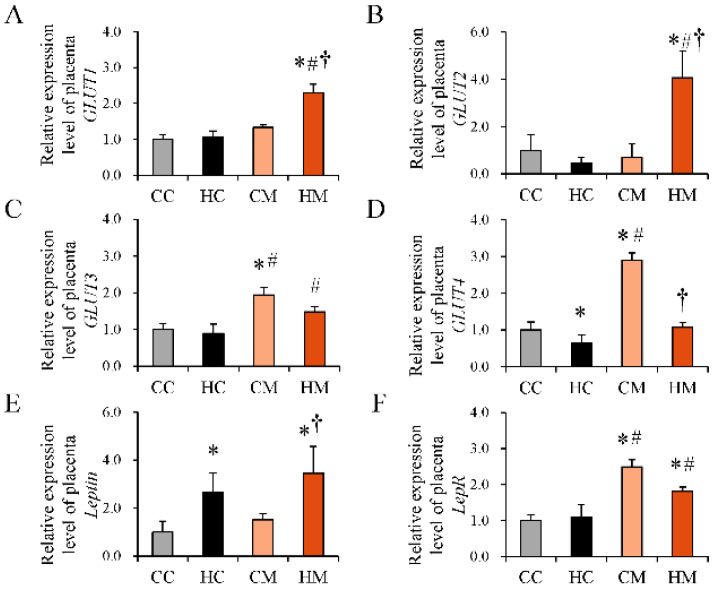
Gene expression of glucose transporters and leptin signaling in placenta tissue. (**A**) GLUT 1, (**B**) GLUT 2, (**C**) GLUT 3, (**D**) GLUT 4, (**E**) leptin, and (**F**) leptin receptor mRNA expression in the placenta tissue of rats administered a maternal control diet (CC), maternal high-fat diet (HC), maternal control diet with metformin treatment (CM), or maternal high-fat diet with metformin treatment (HM). * Compared to the CC group, *p* < 0.05; # compared to the HC group, *p* < 0.05; † compared to the CM group, *p* < 0.05. Abbreviation: GLUT, glucose transporter; LepR, leptin receptor.

We examined the expression of placental GLUT-related genes (Figure 3). We found that exposure of dams to an HF diet decreased the expression of placental GLUT4 (Figure 3D), whereas metformin treatment during pregnancy increased the expression of GLUT1, GLUT2, GLUT3, and GLUT4 mRNA (Figure 3A–D). HF diet-exposed dams receiving metformin treatment during pregnancy showed enhanced mRNA expression of leptin receptor (Figure 3F) but not leptin (Figure 3E). Moreover, maternal metformin treatment alone enhanced the expression of GLUT3, GLUT4, and leptin receptor mRNA as compared with the control group.

### 2.4. Maternal Metformin Treatment Activates 5’ Adenosine Monophosphate-Activated Protein Kinase (AMPK) Signaling in the Placenta

**Figure 4:**
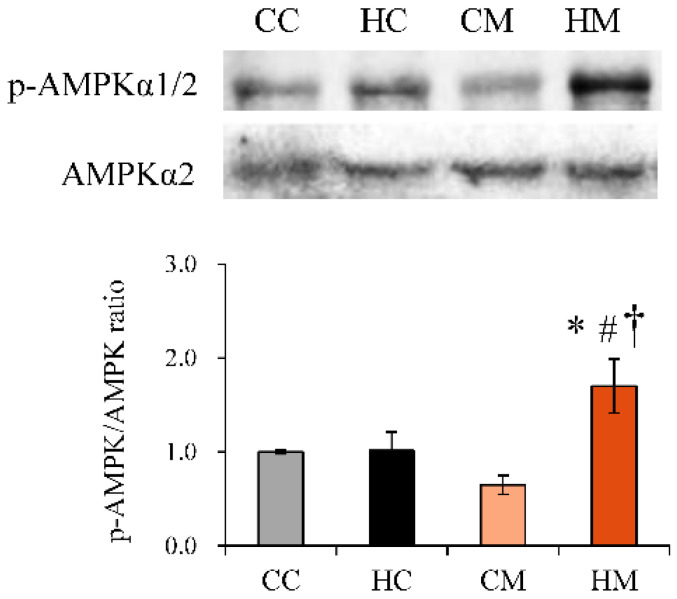
AMPKα2 and AMPKα Thr172 phosphorylation protein abundance in placenta tissue of rats administered a maternal control diet (CC), maternal high-fat diet (HC), maternal control diet with metformin treatment (CM), or maternal high-fat diet with metformin treatment (HM). Representative immunoblots and densitometric quantification of AMPKα2 and phosphor-AMPKα Thr172 protein are presented. Values are mean  ±  SEM. * Compared to the CC group, *p* < 0.05; # compared to the HC group, *p* < 0.05; † compared to the CM group, *p* < 0.05. Abbreviation: AMPK, adenosine monophosphate (AMP)-activated protein kinase.

Metformin is known to exert regulatory effects in the liver, muscle, and adipose tissue metabolism by activating AMPK. We wanted to determine whether metformin exhibited similar effects on the placenta. Treatment of pregnant dams with metformin induced an increase in AMPK phosphorylation compared to dams on an HF diet alone (Figure 4).

### 2.5. Metformin Intake during Pregnancy Prevents Maternal HF Diet-Induced Fetal Hepatic Steatosis and Oxidative Injury in the Offspring

**Figure 5:**
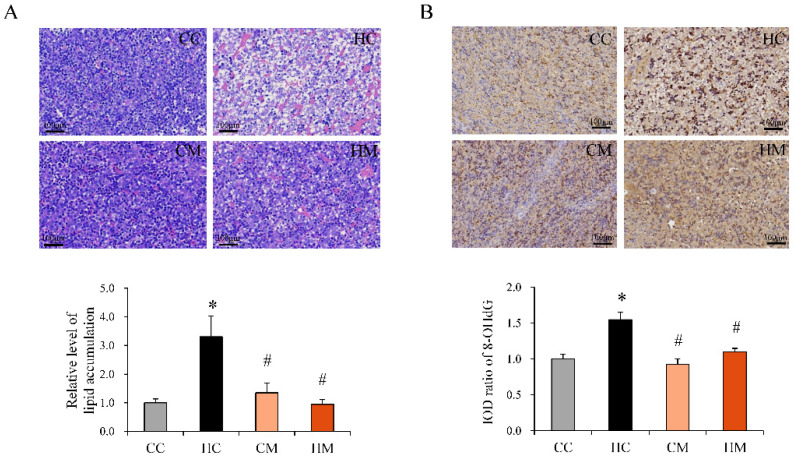
Histological manifestations of the fetal liver with a maternal high-fat diet and prenatal metformin treatment. (**A**) The extent of fetal hepatic steatosis is illustrated as vacuolation in the H&E stain. (**B**) The fetal liver oxidative stress was determined with 8-OHdG staining. Study groups: a maternal control diet (CC), maternal high-fat diet (HC), maternal control diet with metformin treatment (CM), or maternal high-fat diet with metformin treatment (HM). * Compared to the CC group, *p* < 0.05; # compared to the HF group, *p* < 0.05. Abbreviation: 8-OHdG, 8-hydroxy-2-deoxyguanosine; IOD, image optical density.

Nonalcoholic fatty liver disease may start in utero and persist into adulthood [15,16]. Oxidative stress and alteration of key enzymes involved in lipid metabolism are partially responsible for this developmental priming [15]. The therapeutic effects of prenatal metformin on fetal steatosis and oxidative injury were determined. Maternal HF diet exposure resulted in fetal liver steatosis, which was illustrated by an increased proportion of vacuolation upon hematoxylin and eosin (H&E) staining (Figure 5A). Fetal fatty liver changes related to prenatal HF diet exposure were ameliorated by maternal metformin intake during pregnancy. Moreover, 8-hydroxy-2-deoxyguanosine (8-OHdG) staining revealed that metformin treatment during pregnancy reduced oxidative stress in the fetal liver, induced by a maternal HF diet (Figure 5B).

### 2.6. Maternal Metformin Administration Improves the Alternation of Key Enzymes Involved in Lipid Metabolism in Fetal Liver

**Figure 6:**
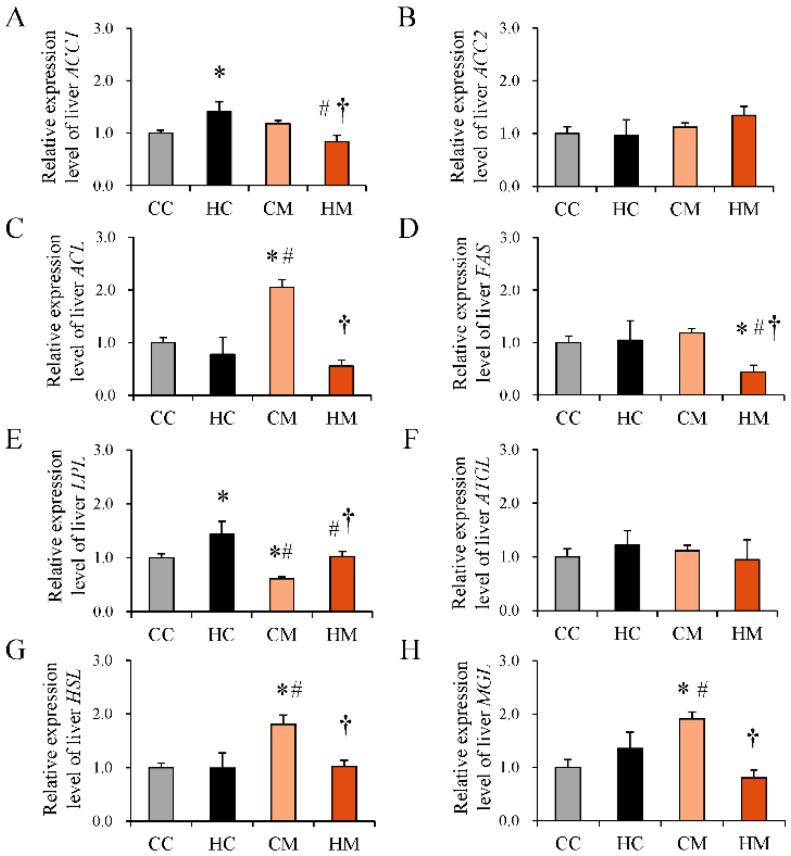
Messenger RNA expression of key enzymes for lipid metabolism in fetal liver. (**A**) ACC1, (**B**) ACC2, (**C**) ACL, (**D**) FAS, (**E**) LPL, (**F**) ATGL, (**G**) HSL, and (**H**) MGL mRNA expression in the fetal liver of rats fed a maternal control diet (CC), maternal high-fat diet (HC), maternal control diet with prenatal metformin treatment (CM), or maternal high-fat diet with prenatal metformin treatment (HC). The housekeeping gene was GAPDH, and mRNA expression is shown as the relative fold. * Compared to the CC group, *p* < 0.05; # compared to the HC group, *p* < 0.05; † compared to the CM group, *p* < 0.05. Abbreviations: ACC1, acetyl-CoA carboxylase 1; ACC2, acetyl-CoA carboxylase 2; ACL, ATP-citrate synthase; LPL, lipoprotein lipase; ATGL, adipose triglyceride lipase; HSL, hormone-sensitive lipase; MGL, monoacylglycerol lipase; GAPDH, glyceraldehyde-3-phosphate dehydrogenase.

Since metformin treatment during pregnancy can reduce fetal fatty liver, we further examined its effects on the gene expression of several important enzymes related to fat metabolism in the fetal liver, including ACC1, ACC2, ACL, FAS, LPL, ATGL, HSL, and MGL (Figure 6). Maternal HF diet exposure led to higher ACC1 and LPL mRNA expression in the fetal liver than chow diet exposure. Compared with the HF group, the HM group showed lower expression of ACC1, FAS, and LPL mRNA in the fetal liver. Maternal metformin treatment alone enhanced the expression of ACL, HSL, and MGL but suppressed LPL mRNA in the fetal liver as compared with the control group.

### 2.7. Changes in Hepatic Fatty Liver in the Offspring That Are Programmed by Maternal HF Diet Are Sustained into Adulthood and Are Reprogrammed upon Maternal Metformin Treatment

**Figure 7:**
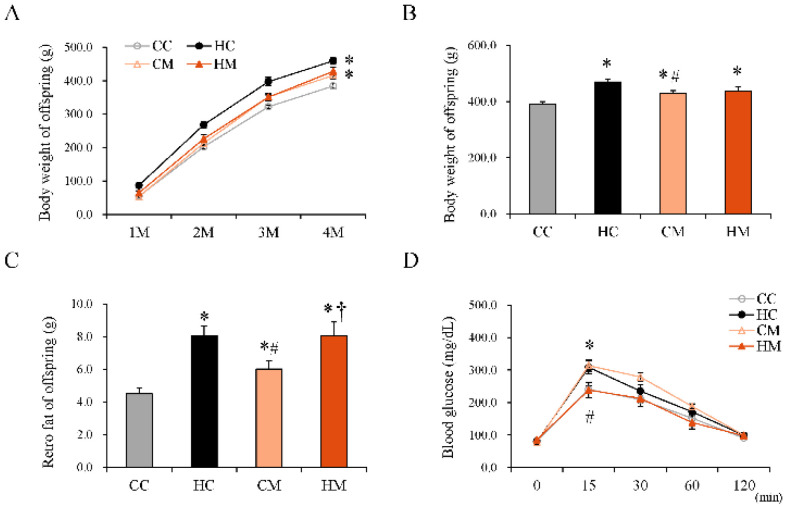
Physiological characteristics of 120-day-old offspring fed a maternal control diet (CC), maternal high-fat diet (HC), maternal control diet with maternal metformin treatment after mating (CM), or maternal high-fat diet with maternal metformin treatment after mating (HM). (**A**) Body weight (BW) change; all the offspring received a control diet after weaning. A significant increase in BW was observed at 4 months of age. Detailed BW data are presented in Supplementary Table S4. (**B**) BW of offspring at 120 days of age. (**C**) Weight of retroperitoneal fat depot. (**D**) Intraperitoneal glucose tolerance test (IPGTT) data. Detailed blood sugar and area under the curve (AUC) data are presented in Supplementary Table S5. The results are presented as mean ± standard error. * Compared to the CC group, *p* < 0.05; # compared to the HC group, *p* < 0.05; † compared to the CM group, *p* < 0.05.

**Figure 8:**
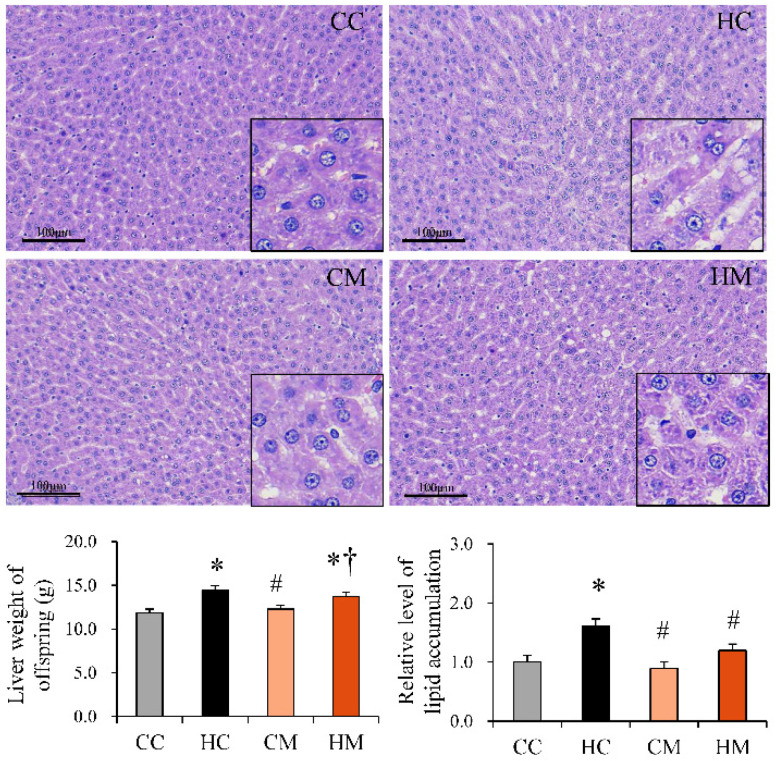
Liver weight and histological manifestations of 120-day-old offspring fed a maternal control diet (CC), maternal high-fat diet (HC), maternal control diet with maternal metformin treatment after mating (CM), or maternal high-fat diet with maternal metformin treatment after mating (HM). All the offspring received a control diet after weaning. The degree of hepatic steatosis is presented as a vacuolation increase via hematoxylin and eosin (H&E) stain. Magnification of boxed area detailing the hepatocytes. * Compared to the CC group, *p* < 0.05; # compared to the HC group, *p* < 0.05; † compared to the CM group, *p* < 0.05.

To test the reprogramming effect of maternal metformin treatment on offspring with maternal HF exposure, another experiment was conducted on offspring fed a maternal control diet or HF diet with or without maternal metformin therapy until delivery. All offspring of these four groups received a chow diet after weaning, until 4 months old (adulthood), and their BWs were observed. In addition, they were evaluated by IPGTT and histologic study. The BWs of offspring each month (Figure 7A) and at 4 months (Figure 7B) are indicated. The BWs of offspring fed a maternal HF diet were heavier than those fed a maternal control diet (HC vs. CC; 459.63 ± 10.85 vs. 384.15 ± 7.22 Kcal/g; *p* < 0.05). Upon maternal metformin treatment, the BWs of the offspring tended to be lower than that of the HC group, although the difference was not statistically significant. Maternal metformin treatment did not alter the retroperitoneal depot weight of the offspring (Figure 7C). In the IPGTT, adult offspring of the HM group showed lower glucose levels at 15 min than adult offspring of the HC group, but the change in AUC did not show statistical significance (Figure 7D). Histological evaluation of livers from offspring in adulthood showed increased proportion of vacuolation, upon H&E staining, in the maternal HF diet-exposed group compared with that in the control group. Changes in hepatic fat in the offspring that are programmed due to a maternal HF diet can be reprogrammed by maternal metformin treatment during pregnancy (Figure 8).

## 3. Discussion

Obesity and HF diet exposure in pregnant women are strongly associated with obesity and metabolic problems in the offspring [15,17,18,19,20]. A maternal HF diet during pregnancy alters key enzymes involved in lipid metabolism and results in steatosis of the fetal liver [15], whose effects can persist into adulthood, even though the offspring received a normal diet after weaning. The programming effect of a maternal HF diet is related to placental remodeling. In this study, we found that metformin treatment, during pregnancy, ameliorated obesity, visceral adiposity, and hepatic steatosis in dams exposed to an HF diet. In addition, maternal metformin intake during pregnancy prevented fetal hepatic steatosis and in addition, reprogrammed changes induced in the fatty liver in adulthood due to prenatal HF diet exposure. Metformin administration during pregnancy also decreases placental oxidative stress and modulates GLUT gene expression. The modification of key enzymes involved in lipid metabolism in the fetal liver might be responsible for the reprogramming effect of maternal metformin therapy.

As a hypoglycemic agent, the primary mechanism of action of metformin is to reduce glucose production in the liver and intestinal absorption of glucose, and increase insulin sensitivity in order to lower blood glucose levels [21]. The most well-known molecular mechanism is the activation of the AMPK pathway, with evidence also suggesting that metformin can regulate AMPK independent pathways such as autophagy and oxidative stress [22]. Although it is not officially approved as a weight-loss drug by the US Food and Drug Administration, many studies support the use of metformin for weight loss in obese patients [23]. In addition, it has many other advantages, including improved insulin resistance in obese patients. However, the weight loss effects of metformin have been inconsistent across reports [24,25]. Although the data is limited, a meta-analysis demonstrated that high-dose metformin treatment can moderately reduce body mass index (BMI), especially for simple obesity and those with a BMI > 35 kg/m^2^ [26]. There are very few studies that report the association of metformin with weight loss in pregnant women. The results of a single-center retrospective cohort study that enrolled pregnant women with type 2 diabetes or prediabetes showed that metformin exposure during pregnancy was associated with less excess weight gain and a higher proportion of weight reduction [27]. This study suggests that the use of metformin in pregnant women may help in avoiding excess weight gain and its associated comorbidities. Our results also showed that metformin treatment during pregnancy reduced obesity and adiposity in dams fed an HF diet. A previous study reported no apparent teratogenic effects, intrauterine death, or fetal growth retardation upon metformin use during pregnancy in women with polycystic ovary syndrome [28]. Furthermore, there were no obvious gestational side effects observed in our study.

In addition to its hypoglycemic effect, metformin can inhibit lipogenic enzymes, especially acetyl-CoA carboxylase (ACC), an important rate-controlling enzyme involved in the synthesis of malonyl-CoA, by activating AMPK in the liver tissue [21]. Metformin reduces lipogenesis through lipogenic enzyme inhibition and promotes fatty acid oxidation, thereby reducing triglycerides, cholesterol, and fatty liver. Metformin treatment has been shown to reduce HF diet-induced changes in the fatty liver in pregnant and non-pregnant rodents [29,30]. In humans, the therapeutic effect of metformin on the fatty liver has not been as dramatic as that observed in rodent studies, as indicated by liver histology. A systematic review of six randomized controlled trials examining the efficacy of metformin in patients with nonalcoholic fatty liver disease reported that it ameliorated serum liver enzyme levels but failed to improve the histological features [31].

Studies on the effect of maternal metformin exposure on the livers of offspring are few. A previous study investigated the effects of maternal metformin intervention during pregnancy in C57BL/6J mice on an obese diet. Female offspring with prenatal metformin exposure exhibited increased hepatic lipid accumulation compared to those without prenatal metformin treatment at 12 months of age [32]. Another study using Wistar rats showed that consumption of a high-calorie diet reduced anti-inflammatory polyunsaturated fatty acids and induced inflammation in the fetal liver. Maternal metformin treatment attenuates some high-calorie diet-induced changes in fatty acids and inflammation in the fetal liver [33]. Our previous study found that post-conception administration of metformin in Sprague–Dawley (SD) rats ameliorated maternal HF diet-induced fetal fatty liver, intestinal inflammation, and apoptosis [29]. Here, we further showed that maternal metformin intake during pregnancy partially restored the changes in fetal liver fatty acids and altered the enzymes involved in lipid metabolism. Maternal metformin intervention during pregnancy even reprograms maternal HF diet-induced changes in the fatty liver in adulthood.

GLUTs are a group of membrane proteins that facilitate glucose transport across the plasma membrane. These transporters are important since glucose is the energy source for all cells, and they control glucose uptake to provide a fine-tuned balance for glucose metabolism and signal production, thereby maintaining cellular metabolic integrity. Based on sequence similarity, the 14 GLUT proteins can be divided into three classes [34]. Decreased glucose transport activity leads to abnormal use of energy substrates and is associated with insulin resistance [35]. GLUTs 1–4 are the most well-known isoforms, with distinct modulatory properties that reflect their specific roles in cellular and systemic glucose homeostasis [36]. Glucose transport in the placenta is important because the placenta provides glucose to the growing fetus. Glucose transport needs to be adjusted in response to the changing metabolic demands of the placenta. The performance of the placental glucose transport system changes with the progression of pregnancy and the developmental and metabolic status of the fetus. In agreement with the data reported by Zhao et al. [37], the placenta of pregnant mice fed an HF diet showed decreased GLUT4 mRNA expression. By contrast, we did not observe a higher expression of GLUT1 mRNA in the placenta of HF diet-fed dams than that in the control diet-fed group. This inconsistent result was most likely due to the differences in species or experimental design.

Metformin has been reported to activate AMPK and enhance the transcription and translation of the insulin-dependent glucose transporter GLUT4 in the endometria of patients with hyperinsulinemic polycystic ovary syndrome [38]. In an in vitro study, adipocytes treated with metformin activated AMPKα1, thereby increasing GLUT4 mRNA and protein abundance, resulting in increased glucose uptake [39]. In this study, we found that maternal metformin treatment enhanced placental class I GLUT mRNA expression through AMPK activation. This novel finding regarding the effect of metformin in the placenta has not been reported in the literature, and it provides an advanced understanding of the multi-organ functions of metformin.

However, there are several limitations of our study. First, the results obtained from offspring are essentially linked to the use of groups of animals different from those used for the analysis of the placenta and fetus. Second, only 8-OHdG was used as a marker of oxidative damage in this study. The use of another marker to examine oxidative stress can strengthen our findings.

In conclusion, our study showed that metformin treatment during pregnancy ameliorates placental oxidative stress and enhances placental class I GLUT expression through AMPK activation. Maternal metformin treatment could reprogram maternal HF diet-induced changes in offspring fatty liver, with the effects observed in adulthood as well. Further validation is required to develop maternal metformin therapy for clinical applications.

## 4. Materials and Methods

### 4.1. Study Animals and Experimental Design

This study was approved by the Institutional Animal Care and Use Committee of Chang Gung Memorial Hospital (number: 2019053001). Virgin, female Sprague–Dawley rats aged 7 weeks (purchased from BioLASCO Co., Ltd., Taipei, Taiwan) were housed under a 12-h light–dark cycle, at 22 °C, and 55% humidity. Sterile tap water and food were provided ad libitum [20]. The rats were weight-matched and assigned to receive either a regular control diet (27.5% protein, 59.7% carbohydrate, 12.6% fat by energy, 3.25 kcal/g; Fwusow Taiwan Co., Ltd., Taichung, Taiwan) or an HF diet (16.4% protein, 25.5% carbohydrate, 58% fat by energy, 5.24 kcal/g; D12331, Research Diets, Inc., New Brunswick, NJ, USA) after one week of adaptation. The rats were fed the allocated diet for eight weeks and maintained in an environment conducive for mating for three days. The initiation of gestation was confirmed by checking the vaginal plug, and mating day 1 was considered gestational day (GD) 1.

To investigate the therapeutic effect of maternal metformin treatment, rats were divided into four groups: CC group (dams were fed a normal chow diet before and during pregnancy); HC group (dams were fed an HF diet before and during pregnancy); CM group (dams were fed a normal chow diet before and during pregnancy, and metformin (500 mg/kg/day), dissolved in drinking water, was administered between gestational day (GD) 0 and GD 21); and HM group (dams were fed an HF diet before and during pregnancy, and metformin (500 mg/kg/day), dissolved in drinking water, was administered between gestational day (GD) 0 and GD 21) (Supplementary Figure S2). To determine the preventive effect of maternal metformin treatment, dams were sacrificed on GD 21 after 8 h of fasting before delivery of pups, and dam and fetus samples were collected. To determine the reprogramming effect of maternal metformin treatment, offspring were fed a chow diet after weaning and sacrificed on GD 120 (adult stage) after 8 h of fasting in another experiment. The offspring of each group were obtained from a different litter.

### 4.2. Specimen Collection

Rats were sacrificed using a mixture of zoletil (25 mg/kg) and xylazine (23 mg/kg). Heparinized blood samples were collected by cardiac puncture [40]. The liver and retroperitoneal adipose depots of the dams were collected [41]. Placenta and fetal liver were collected via cesarean section. A part of the placenta was fixed in 10% formalin and subjected to histological analysis. Other tissues were stored at −80 °C for quantitative polymerase chain reaction (qPCR) analysis or western blotting.

### 4.3. Body Weight and Blood Pressure Determination

The BWs of dams were measured weekly from 7 weeks of age until the day of sacrifice. The blood pressure (BP) of dams was measured before sacrifice by the indirect tail-cuff method (BP-2000, Visitech Systems, Inc., Apex, NC, USA) [41]. In the reprogramming study, the BW of the offspring was measured after weaning and monthly until sacrifice.

### 4.4. Intraperitoneal Glucose Tolerance Test

An Intraperitoneal glucose tolerance test (IPGTT) was performed on the rats one week before sacrifice. The test was conducted by injecting 50% glucose (2 g/kg body weight) after 8 h fasting as reported previously [15]. Serum glucose levels were measured via the tail vein, using a glucometer (Accu-Chek, Roche, Germany), before injection and 15, 30, 60, and 120 min after injection. The integrated area under the curve (AUC) values of IPGTT were determined.

### 4.5. Histological Study

A Leica RM255 microtome (Leica Biosystems, Concord, ON, Canada) was used to cut the formalin-fixed tissues. The cut sections were scanned using a 3DHISTECH panoramic scanning slide scanner and analyzed using Panorama Viewer software after staining with hematoxylin and eosin (H&E). Scanned images were further analyzed using the ImageJ software, as indicated. Oxidative stress in both placenta and liver tissues was measured using 8-OHdG [42].

### 4.6. RNA Isolation and qPCR Analysis

After RNA isolation [43], messenger RNA (mRNA) expression was further analyzed by qPCR [41,44]. The comparative threshold cycle method was used for the relative quantification of gene expression. The primer sequences used are listed in Supplementary Table S1.

### 4.7. Western Blot Analysis

Western blot analysis was performed as described previously [45]. Briefly, total protein was isolated using protein extraction reagents (iNtRon Biotechnology, Sungnam, Korea). Protein concentration was determined using the BCA protein assay kit (Pierce, Rockford, IL, USA). An equal amount of protein (50 μg) from each sample was used for electrophoresis. After electrophoresis on a 12% sodium dodecyl sulfate-polyacrylamide gel, the fractionated proteins were transferred onto a polyvinylidene difluoride membrane. After blocking with TBST (Tris-buffered saline with 0.10% Tween 20) containing 5% nonfat milk, the membrane was first incubated overnight at 4 °C with anti-phospho-AMPKα1/α2 (Thr172) (sc-33524, Santa Cruz Biotechnology, Santa Cruz, CA, USA) antibody, stripped, and then incubated with anti-AMPKα2 (sc-19129, Santa Cruz Biotechnology) antibody (1:500). Subsequently, the membrane was incubated with peroxidase-conjugated secondary antibody diluted 1:1000. The signal was obtained using the Bio-Rad Molecular Imager ChemiDocMP and quantified using Image Lab version 5.0 software (Bio-Rad, Hercules, CA, USA). The individual values were originally expressed as a ratio of phospho-AMPKα1/α2 to total AMPKα2, and then expressed as a fold change compared to the CC group.

### 4.8. Statistics

One-way analysis of variance (ANOVA) was performed to compare the differences between different groups. Bonferroni correction was used to determine the subsequent sample effects. Values are expressed as mean ± standard error of the mean, and results with a *p*-value of <0.05 were considered statistically significant. All statistical analyses were performed using SPSS 22.0 for Windows XP (SPSS, Inc., Chicago, IL, USA).

## 5. Conclusions

In conclusion, our data suggest that prenatal intervention with metformin may partially reprogram maternal HF diet-induced hepatic fatty changes in the offspring, which continues until the adult stage. In the placental tissue, maternal metformin treatment decreases placental oxidative stress, activates AMPK signaling, and augments *GLUT* expression. Furthermore, maternal metformin treatment modulates key enzymes related to lipid metabolism in the fetal liver.

## Data Availability

Data are available upon request from the corresponding author after publication.

## Supplementary Materials

The following supporting information can be downloaded at: https://www.mdpi.com/article/10.3390/ijms232214239/s1.
