# Supplementary material for: Maternal Metformin Treatment Reprograms Maternal High-Fat Diet-Induced Hepatic Steatosis in Offspring Associated with Placental Glucose Transporter Modifications
Source: Int J Mol Sci. 2022 Nov 17;23(22):14239. doi: 10.3390/ijms232214239 (PMC9694630; doi:10.3390/ijms232214239)
Supplement: Supplementary file 1 [file ijms-23-14239-s001.zip › ijms-2014685-supplementary.pdf]

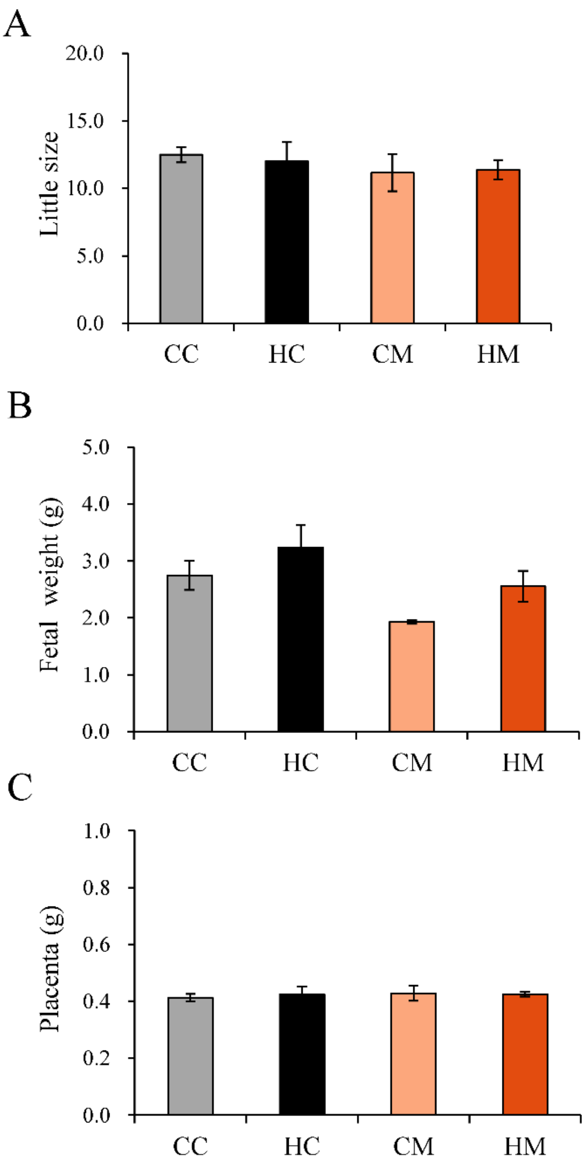

Figure S1: The placental weight and litter characteristics among the four groups (A) little size (B) fetal weight (C) placenta weight.

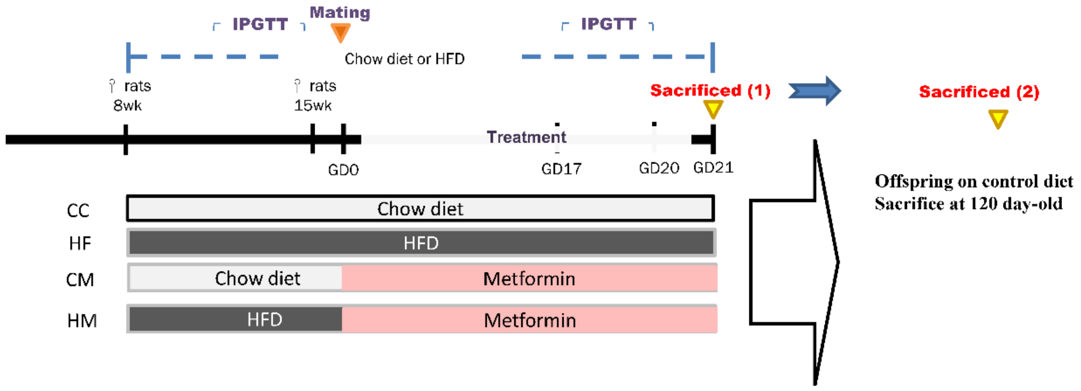

Figure S2: Schematic diagram of the experimental design.

Supplementary Table S1. Primer sequences for RT-PCR in this study.

Rat Gene	primer	sequence (5' to 3')
GLUT1	Forward	TGGCCAAGGACACACGAATACTGA
	Reverse	TGGAAGAGACAGGAATGGGCGAAT
GLUT2	Forward	TAGTCAGATTGCTGGCCTCAGCTT
	Reverse	TTGCCCTGACTTCCTCTTCCAACT
GLUT3	Forward	TGGCTACAACACCGGAGTCATCAA
	Reverse	CTGCCAAAGCGGTTGACAAAGAGT
GLUT4	Forward	TTTCCAGTATGTTGCGGATG
	Reverse	TCAGTCATTCTCATCTGGCC
Leptin	Forward	CGGTTCCTGTGGCTTTGGT
	Reverse	CCGACTGCGTGTGTGAAATG
LepR	Forward	ACTGGGACATAGAGTGCTGGAT
	Reverse	GTTGCACTGGACAGTCTGAA AG
ACC1	Forward	TGAGGAGGACCGCATTTATC
	Reverse	GCATGGAATGGCAGTAAGGT
ACC2	Forward	CGCTGCGGTCAAGTGT
	Reverse	CGTTGGCGTAGTTGTTATT
ACL	Forward	ACCCAGAGGAAGCCTACATTGC
	Reverse	TTCGCCAGTTCGTTGACACC
FAS	Forward	AGATCCTGGAACGTGAACATGA
	Reverse	GCCGTACTTCACGAATGGGT
LPL	Forward	GTACAGTCTTGGAGCCCATGC
	Reverse	GCCAGTAATTCTATTGACCTTCTTGTT
ATGL	Forward	TGTGGCCTCATTCCTCCTAC
	Reverse	TCGTGGATGTTGGTGGAGCT
HSL	Forward	GCTGGGCTGTCAAGCACTGT
	Reverse	GTAACTGGGTAGGCTGCCAT
MGL	Forward	TGACCAACTCTGTCCTCCAT
	Reverse	GCACTGCCCTTCCTCTT
18S	Forward	GCGATGCGGCGGCGTTAT
	Reverse	AGACTTTGGTTTCCCGGAAGC
GAPDH	Forward	TCTTGTGCAGTGCCAGCCTC
	Reverse	GTCACAAGAGAAGGCAGCCCTGG

Abbreviation: GLUT1, glucose transporter 1；GLUT2, glucose transporter 2；GLUT3, glucose transporter 3；GLUT4, glucose transporter 4；LepR, leptin receptor；ACC1, acetyl-CoA carboxylase 1；ACC2, acetyl-CoA carboxylase 2；ACL, acylcholesterol

lipase; FAS, fatty acid synthetase；LPL, lipoprotein lipase; ATGL, adipose triglyceride lipase；HSL, hormone-sensitive lipase；MGL, monoacylglycerol lipase；GAPDH, glyceraldehyde-3-phosphate dehydrogenase

Supplementary Table S2. The weekly body weight change of dams

	8w	9w	10w	11w	12w	13w
**CC**	210.22±3.33	208.80±2.68	214.90±2.61	227.55±2.95	231.84±2.77	240.28±2.93
**HC**	217.21±4.17	234.51±5.59*	255.14±8.26*	272.96±8.95*	287.96±9.84*	298.01±9.88*
**CM**	205.38±2.68	199.85±1.94#	205.90±1.73#	217.40±2.42#	225.60±1.87#	232.00±2.19#
**HM**	215.01±4.30	232.80±4.45*†	253.25±4.64*†	269.21±5.36*†	279.28±5.62*†	289.60±7.40*†

	14w	15w	16w	17w	18w	19w
**CC**	252.42±3.03	247.00±4.24	255.40±4.75	276.89±4.81	292.05±5.39	312.47±2.52
**HC**	307.70±10.34*	305.11±11.70*	309.78±13.08*	318.33±18.20*	355.29±11.52*	395.29±10.92*
**CM**	241.03±2.48#	253.07±2.36#	261.47±2.37#	265.25±2.29#	278.15±4.83#	302.00±9.24#
**HM**	298.10±8.39*†	300.75±8.40*†	303.00±8.91*†	291.44±7.99	292.95±6.91#	300.19±6.25#

The results are presented as mean ± standard error. ;*compared with CC, P <0.05; ＃compared with HC, P <0.05; † compared to the CM group, P< 0.05.

Abbreviation: CC, maternal control diet；HC. maternal high-fat diet；CM, maternal control diet plus metformin treatment；HM, maternal high-fat diet plus metformin treatment

Supplementary Table S3. Intraperitoneal glucose tolerance test (IPGTT) of dams

Min.	**CC**	**HC**	**CM**	**HM**
0	71.00 ± 4.70	64.40 ± 4.23	61.60 ± 4.70	57.20 ± 2.27
15	183.80 ± 39.97	386.20 ± 57.93*	290.40 ± 24.06	254.80 ± 49.53*#
30	166.00 ± 30.68	319.20 ± 48.24*	206.60 ± 6.80 #	220.80 ± 48.94
60	114.00 ± 20.71	142.00 ± 14.20	90.00 ± 3.45	127.80 ± 31.18
120	82.80 ± 9.96	76.20 ± 6.87	69.00 ± 6.28	58.40 ± 6.26
AUC (x1000)	14.64 ± 1.76	22.13 ± 2.10	15.59 ± 0.54	16.72 ± 2.70

The results are presented as mean ± standard error. ;*compared with CC, *P* <0.05; ＃compared with HC, *P* <0.05.

The integrated AUC values of the IPGTT were calculated using the trapezoidal method and a repeated-measure analysis of variance (ANOVA) model.

Abbreviation: CC, maternal control diet；HC. maternal high-fat diet；CM, maternal control diet plus metformin treatment；HM, maternal high-fat diet plus metformin treatment；AUC, area under curve

Supplementary Table S4. The monthly body weight change of offspring

	**CC**	**HC**	**CM**	**HM**
1M	54.36±2.38	86.92±3.67*	53.53±1.19#	64.37±6.30*#†
2M	202.40±5.09	268.04±8.48*	213.09±4.01#	226.20±11.92*#
3M	322.32±4.92	397.19±12.38*	351.55±6.11*#	351.31±10.65*#
4M	384.15±7.22	459.63±10.85*	416.15±9.61*#	427.92±12.89*

* compared to the CC group, *P*< 0.05; # compared to the HC group, *P*< 0.05; † compared to the CM group, *P*< 0.05.

Abbreviation: CC, maternal control diet；HC. maternal high-fat diet；CM, maternal control diet plus metformin treatment；HM, maternal high-fat diet plus metformin treatment；AUC, area under curve

Supplementary Table S5. Intraperitoneal glucose tolerance test (IPGTT) of offspring

Min.	**CC**	**HC**	**CM**	**HM**
0	80.92 ± 2.33	80.75 ± 2.97	78.00 ± 2.24	85.75 ± 1.87
15	240.31 ± 18.47	308.58 ± 20.63 *	313.85 ± 16.99 *	238.75 ± 24.33 #†
30	209.46 ± 14.75	235.33 ± 19.30	278.77 ± 12.07 *	213.00 ± 25.33 †
60	152.15 ± 8.62	171.83 ± 9.00	188.23 ± 8.08 *	138.50 ± 20.03 #†
120	90.92 ± 4.89	99.33 ± 6.06	99.69 ± 4.59	98.13 ± 4.08
AUC (x1000)	18.50 ± 0.92	21.24 ± 1.07	23.03 ± 0.84 *	18.19 ± 1.70 †

The results are presented as mean ± standard error. ;*compared with CC, P <0.05; ＃compared with HF, P <0.05; † compared to the CM group, P< 0.05.

Abbreviation: CC, maternal control diet；HC. maternal high-fat diet；CM, maternal control diet plus metformin treatment；HM, maternal high-fat diet plus metformin treatment；AUC, area under curve
